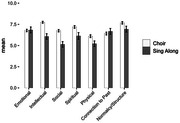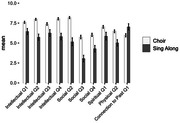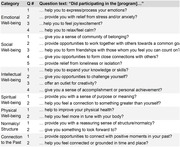# Group Singing Programs for Well‐being of Older Adults: An In‐Person Quantitative Follow‐up

**DOI:** 10.1002/alz.093587

**Published:** 2025-01-09

**Authors:** Borna Bonakdarpour, Rhiana Schafer, Jonathan Miller, Sandra Siegel Miller

**Affiliations:** ^1^ Mesulam Center for Cognitive Neurology and Alzheimer Disease, Chicago, IL USA; ^2^ Northwestern University, Chicago, IL USA; ^3^ University of Chicago, Chicago, IL USA; ^4^ Sounds Good Choir, NFP, Downers Grove, IL USA

## Abstract

**Background:**

Singing improves mood, social, and physical well‐being (Pentikainen et al., 2021). Choral singing has therefore gained recognition as a highly recommended activity for older adults and persons with dementia to fight isolation (Petrovsky et al., 2020; Sakamoto et., 2023). In a previous study, using qualitative measures, we demonstrated virtual sing‐along and choral singing offered by the Chicago‐based Sounds Good Choir and were associated with improved anxiety, emotional, and physical well‐being (Schafer et al., 2023) using qualitative, but not fully quantitative measures. The goal of this study was to determine whether in‐person choral singing and virtual sing‐along programs would improve older‐adults well‐being as measures by 7 quantitative surveys.

**Methods:**

Participants were aged 55 and older. The sing‐along program engaged participants in virtual singing‐along to familiar music weekly for a year. The choir program consisted of 14 in‐person rehearsals to learn different parts of choral pieces. Following the program, self‐report assessments were solicited via 21 Likert scale questions covering 7 areas of well‐being (Table 1). Questions were developed from previous program evaluations and modeled off existing validated assessments of well‐being (Topp et al, 2015; Barrett & Murk, 2006; Dunn, 2008). Data were compared across program types using ANOVA, with Bonferroni corrected post‐hoc pairwise *t* test comparisons.

**Result:**

Participants reported improvements in all aspects of well‐being in both programs (Figure 1). Two areas improved across the board with no effect of program type ‐ *normalcy/structure* and *emotional*. ANOVA indicated four well‐being areas were improved significantly more in choir participants than sing‐along (Figure 2) ‐ *social* (Q2‐4, p<0.001), *intellectual* (Q1‐4, p<0.001), physical (Q2, p<0.01), and *spiritual* (Q1, p<0.01). *Connection to past* was endorsed significantly more by sing‐along participants (p<0.01).

**Conclusion:**

Both choral and sing‐along programs improved all aspects of well‐being with choral singing showing more effect in social, intellectual, physical, and spiritual well‐being, and sing‐along achieving more connection to the past. Our results provide preliminary evidence that our quantitative measures can capture participants’ experiences. While a larger controlled study is necessary for confirmation, our findings may suggest that depending on feasibility, either program may be utilized for measurable well‐being effects in older adults.